# Lifestyle modifications as therapy for medication refractory post-traumatic headache (PTHA) in the military population of Okinawa

**DOI:** 10.1186/s10194-018-0943-2

**Published:** 2018-11-22

**Authors:** Virginia B. Baker, Kathryn M. Eliasen, Nawaz K. Hack

**Affiliations:** 1 Department of Neurology, Walter Reed National Military Medical Center, 8901 Rockville Pike, Bethesda, MD 20889 USA; 2 Department of Anesthesiology, Walter Reed National Military Medical Center, 8901 Rockville Pike, Bethesda, MD 20889 USA

**Keywords:** Post-traumatic headache (PTHA), Chronic post-traumatic headache (CPTHA), Lifestyle, Polypharmacy

## Abstract

**Objective:**

Our aim was 1) to reduce disability, as characterized by headache frequency, duration and severity in patients with post-traumatic headache (PTHA), 2) to reduce the number of medical boards and work limitations in patients with post traumatic headache, and 3) to reduce use of medical resources and clinic visits related to headache or migraine.

**Background:**

Modifiable risk factors for PTHA include stressful life event, sleep disturbances, and medication overuse. Cognitive-behavioral strategies, biofeedback, and relaxation therapy may have an important role in treatment and preventing progression to chronic post-traumatic headache (CPTHA). There is limited literature and a known practice gap for implementation of these techniques.

**Design/methods:**

An IRB approved project focused on patients who were seen for PTHA and CPTHA. 1) Intervention consisted of lifestyle teaching, cognitive-behavioral therapy and biofeedback, supplemented by decreasing polypharmacy. 2) Patients were followed for 2 years and a retrospective review was conducted for 2 years prior to presentation. 3) Outcome measures included reduction in migraine intensity or frequency, improved quality of life, duty status, and decreased utilization of clinic visits.

**Results:**

Over the course of one year, 221 patients were treated for migraines in the Naval Okinawa Neurology Clinic. Of these, 22 active duty service members and 3 Dependents suffered a mild TBI prior to onset. After intervention, there was a 36% decrease in PTHA frequency, 56% decrease in headache severity and 60% of patients had improved quality of life as compared to the 2 years prior to intervention. Twenty-four percent had reduction in polypharmacy. Appointment frequency for migraine decreased from an average of 6.8 to 2.6 per year.

**Conclusions:**

An implemented program geared towards reducing polypharmacy was shown to improve safety, quality of life and reduce hospitalizations from the burden of migraines. Our systematic approach resulted in quality of life improvements and decreased use of medical resources.

**Trial registration:**

Authors received the approval of NAVMED West, Okinawa Naval Hospital Institutional Review Board on January 13th, 2016. QI.2016.0021.

## Background

Traumatic brain injury (TBI) affects 1.7 million Americans and results in 275,000 hospitalizations per year. Among this population, chronic pain has a prevalence of nearly 40%, the majority due to headaches [1–4]. The International Classification of Headache Disorders, edition 3, defines post-traumatic headache (PTHA) as developing within 7 days of injury to the head. This is further defined as acute versus chronic post-traumatic headache (CTPHA) based on persistence after 3 months [5]. In active duty servicemen with a history of TBI, one study documented nearly 20% had chronic daily headache and 78% had episodic headache [6]. A review of headache after deployment-related TBI noted an even higher occurrence of chronic daily headache, occurring in 44% as compared to 7% of patients who suffered head injury not related to deployment. The progression to chronic migraine is 4–5 times more frequent in the military population, likely due to known psychosocial stressors associated with service. This is compounded by limited preventative measures and lifestyle interventions received in a deployed setting [7]. History of concussion or mild TBI has been shown to correlate with poor mental health outcomes, particularly for those who endorse PTHA [8–11]. Furthermore, it is recognized that psychiatric symptoms can make recovery more difficult [12].

Relaxation training, counseling, cognitive behavioral therapy (CBT) and behavioral therapy have demonstrated efficacy in small controlled trials and retrospective case reports in treatment of post-traumatic headache disorders [8, 13, 14]. Two studies demonstrated significant improvement in headache outcomes, general well-being and up to 50% reduction in self-reported headache days with use of CBT or behavioral therapy [14, 15]. A pilot study performing one-day behavioral intervention on patients with headaches and coinciding anxiety or depression demonstrated notable improvement in the absence of new medication [16]. A systematic review of treatment strategies for PTHA ultimately proposed a treatment model that utilizes behavioral therapy in difficult-to-control headache disorders [17]. However, there is a known practice gap for implementation of these techniques in active duty service members with prior head trauma [18–20].

Presently, there are no FDA approved medications for PTHA or CTPHA and there is no strong evidence for a standardized treatment approach [10, 17, 21]. Yet these conditions are often over-medicated with both prescription and over the counter pharmacotherapies [22, 23]. Medication overuse is especially problematic in the military population, and both TBI and PTSD have been shown to increase the risk for overuse [24–26]. Polypharmacy in the setting PTHA leads to greater disability, increases the chance of drug interactions and ultimately increases mortality and suicidal behaviors [27]. A systematic management program that is geared toward reducing polypharmacy in this population can improve patient safety and reduce hospitalizations from the burden of migraines [28].

PTHA presents a special challenge to remote populations with limited access to neurologic and medical care. This is problematic for deployed active duty service members who are expected to perform in any environment [3, 29–32]. Physical limitations are especially consequential, as they can increase use of medical resources, decrease productivity, and ultimately affect the military mission as a whole. We present an active duty and dependent population in the remote Pacific with post-traumatic headache or chronic post-traumatic headache who benefited from a unified multidimensional approach. Our intervention focused on lifestyle modifications, stress-management, and polypharmacy reduction in order to reduce migraine burden, improve quality of life, and decrease healthcare resource expenditure.

## Methods

We collected data using a protocol that retrospectively reviewed a remote Neurology clinic’s systematic approach to treating PTHA and CPTHA. The study was approved through NAVMED West, Okinawa Naval Hospital institutional review board and complied with all ethical guidelines. Our selected population consisted of patients presenting to the Neurology Clinic at Naval Hospital Okinawa between September 2014 and December 2015. Chart review was performed on all patients over age 18 who were active duty or an active duty dependent with a diagnosis of headache or migraine in the electronic medical record (EMR). These diagnoses were made by a single Neurologist at Okinawa Naval Hospital based on Department of Defense (DoD) guidelines. Two hundred twenty-one patients presented to Neurology clinic and were diagnosed with headache or migraine over this timeframe. All patients were required to have persistence of headaches for > 4–6 weeks after initial treatment by a primary care provider or traumatic brain injury specialist. The cohort was further stratified based on development or worsening of headache symptoms within 7 days after mild traumatic brain injury. Mild traumatic brain injury was defined as traumatically induced injury or disruption of brain function with normal structural imaging and less than 30 min loss of consciousness [33]. All patients met criteria for outpatient management of traumatic brain injury and were screened for underlying secondary headache disorder or intracranial complications, in which case they were excluded. At the time of presentation to the Neurology clinic, all service members were facing the possibility of duty restrictions or medical separation.

Over 1 year, patients seen in Neurology clinic for mild TBI, headache or migraine were provided didactics on pathophysiology of migraines, tension-type headaches and on post-traumatic headaches. Didactics discussed the importance of a multidimensional approach to management, focusing on both pharmacotherapy and non-pharmacotherapy options. We discussed the benefits and limitations of medications in treatment of headaches. The importance of lifestyle modification was discussed. This included oral hydration therapy (i.e. one 16-oz electrolyte drink each morning prior to starting work), improvements in sleep hygiene and stress reduction. Patients were encouraged to keep a stable sleep schedule and were instructed to find a relaxing activity 30 min prior to sleep. Stress-reduction was encouraged through cognitive-behavioral therapy, biofeedback and meditation exercises. Each patient was provided a pamphlet summarizing these recommendations which was reviewed with the Neurologist during the clinic visit. Finally, patients were encouraged to engage in reduction of polypharmacy, as able. Down-titration or discontinuation of either abortive or preventative medications used for treatment of headache was guided by the Neurologist. Botulinum toxin was offered as an adjunctive treatment to assist in reduction of polypharmacy, but was only utilized in 2/25 PTHA or CPTHA patients. Pregnant patients were not offered Botulinum toxin, but within the PTHA / CTPHA cohort, there were none.

Patients had follow-up scheduled in Neurology clinic, TBI clinic and with primary care providers for migraine headaches. Other providers within the Okinawa Naval Hospital were educated about TBI and migraines through CME courses, telemedicine, Radio Broadcast shows, and community health fairs lead by the central Neurologist. All physicians were encouraged to advocate for lifestyle modification and polypharmacy reduction in migraine patients. At each Neurology visit, migraine severity, frequency, duration, and updated medication regimen were assessed and subjective quality of life (self-report of patient’s improvement) was documented in the EMR. A patient’s ability to perform at work was evaluated and the decision to medically board or restrict job assignment was made.

Participants in the study consented to implementing lifestyle modifications and were in agreement with the plan to reduce medication regimens as able. All personal information was de-identified in accordance with HIPPA regulations (Health Insurance Portability and Accountability Act of 1996). For PTHA and CPTHA patients, data was retrospectively collected starting 1 month after injury, for 2 years prior to intervention in Neurology clinic. Data collection was from EMR notes at each visit with a documented chief complaint of headache or migraine. It was also prospectively collected 2 years after intervention. Data collected included demographics, migraine frequency, duration and severity, quality of life, current deployment and duty status, and current pharmacotherapy. Medications reviewed included pain medications (i.e. Gabapentin, non-steroidal anti-inflammatories (NSAIDS), and opioids) and headache preventative and abortive pharmacotherapies. The preventative agents included were Topiramate, Nortriptyline, Propranolol, Amitriptyline, Gabapentin, Clonidine, Verapamil, Venlafaxine and Indomethacin. The abortive medications included were Triptan agents, non-steroidal anti-inflammatories (NSAIDS), Naproxen Butalbital/Acetaminophen/Caffeine. Anti-emetics and supplements were excluded from this calculation.

In this study, each patient served as his or her own control. Data was collected and analyzed through a protected version of Excel. Descriptive statistics was used to summarize demographic and clinical characteristics. Chi-squared testing was used to determine statistical significance for categorical variables.

## Results

Data collection included 221 patients who were treated by a single Neurologist over 806 appointments. Of these, 22 active duty service members (AD) and 3 Dependents suffered a mild traumatic brain injury, leading to post-traumatic headaches or chronic-post traumatic headaches. All patients with PTHA or CPTHA had headaches which persisted for greater than 4 weeks after initial injury in order to meet inclusion criteria. Patient’s average age was 29 years old, of predominantly male sex (68%), and primarily active duty (89%). Thirty two percent of patients had CPTHA (headache > 15 days per month). 32% of patients were on 2 or more medications at the time of initial evaluation. Sixty four percent of patients were diagnosed and treated for a coinciding psychiatric disorder, which included adjustment disorder, major depressive disorder, generalized anxiety disorder, or post-traumatic stress disorder (PTSD) (Table 1).

**Table 1:** Demographics of patients enrolled

	LOC (*n* = 13)	NO LOC (*n* = 12)
Average Age	29	28
Male sex	64%	73%
Active duty	93%	82%
Dependent	7%	18%
TBI mech	21% IED Blast, 14% Syncope	18% IED blast, 9% Syncope
% Chronic migraine	43%	18%
>/= 2 meds at initial visit (*p* < .010)	50%	9%
Psychiatric diagnosis (*p* < .010)	79%	45%
Line of duty	64%	55%

**Fig. 1:**
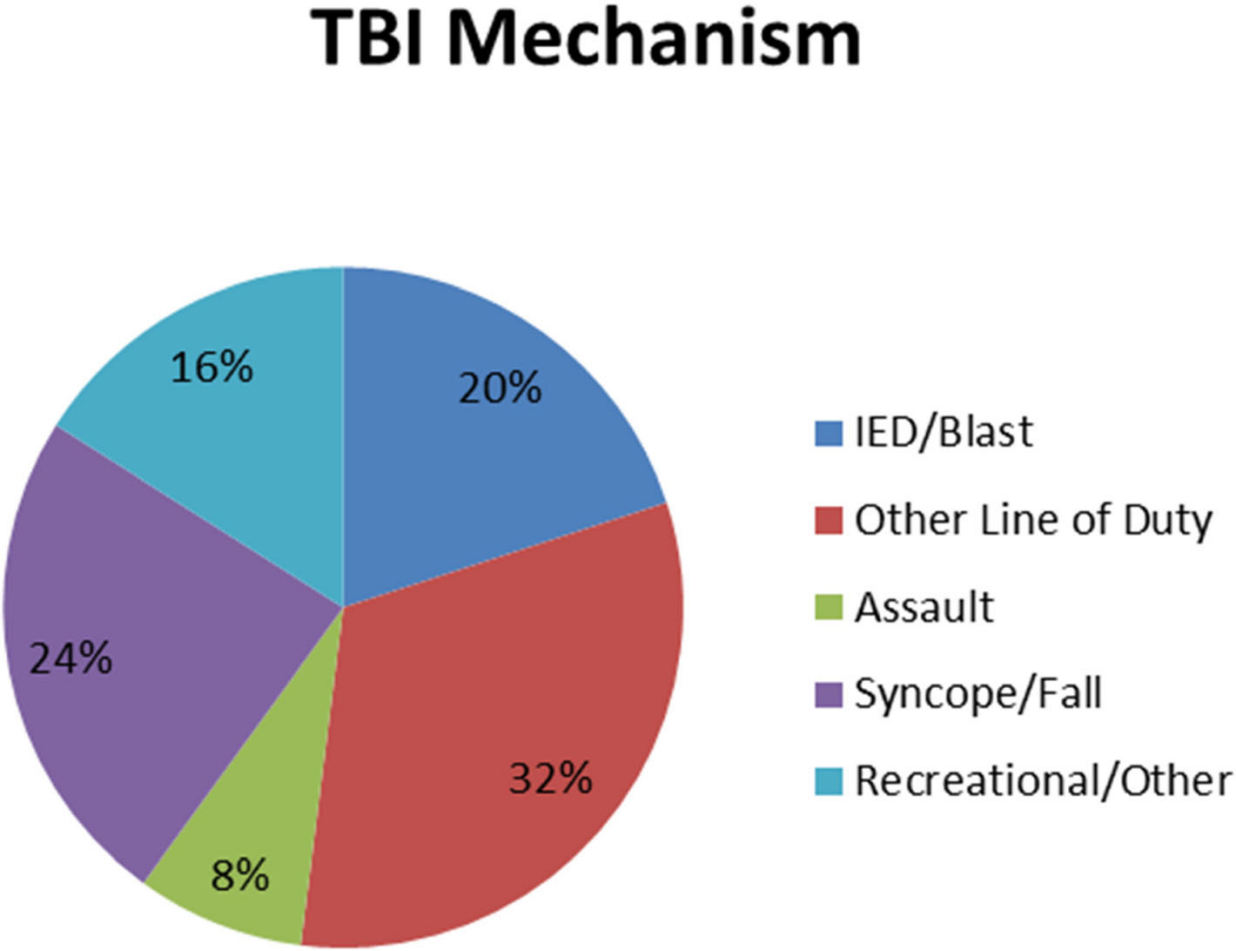
Mechanism for TBI of patients enrolled

All patients included in our demographic sustained a mild traumatic brain injury. TBI occurred 10 years to 1 month prior to intervention. The average time from injury was 26 months. 52% of patients were greater than 1 year and two patients were greater than 10 years from injury. All patients had been treated by other providers for PTHA or CPTHA prior to presentation to the Neurology clinic. Mechanisms for TBI were varied, with 20% from IED or blast, 32% occurring from other trauma associated with line of duty, 24% secondary to fall or syncope, 20% sustained from recreational activity, and 8% from assault (Fig. 1). More than half of injuries involved loss of consciousness. Patients who sustained a mild TBI with loss of consciousness were significantly more likely to be on more than one medication for PTHA or CPTHA at time of presentation. They were also more likely to have psychiatric comorbidities.

**Fig. 2:**
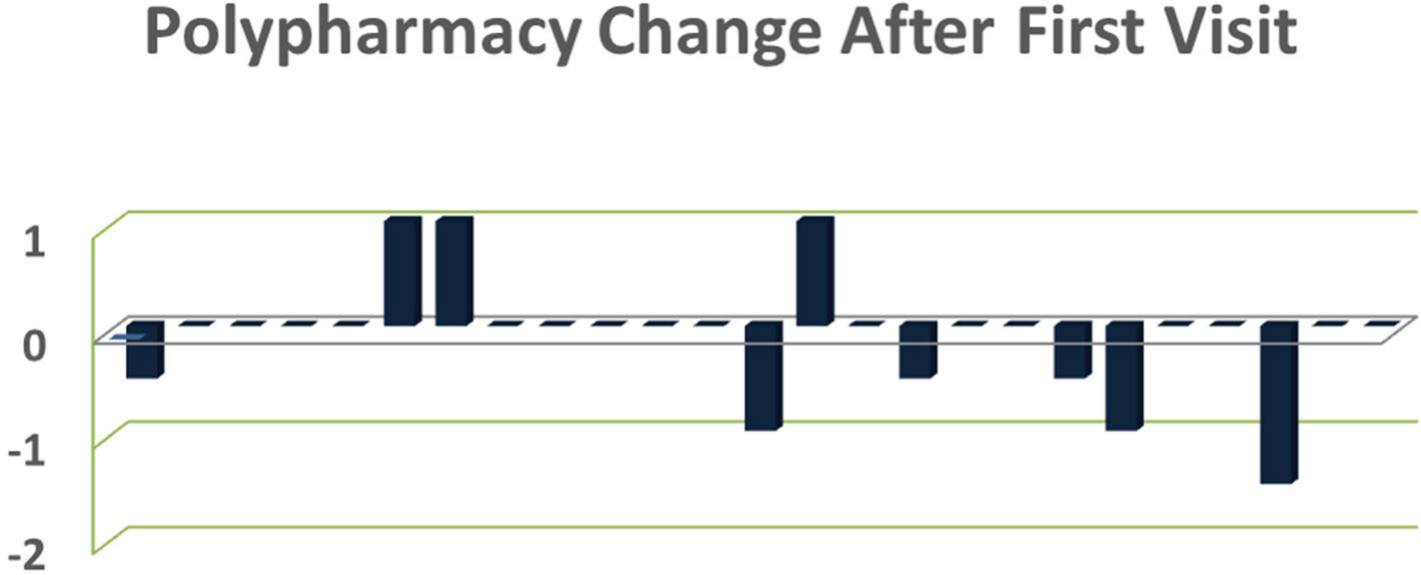
Medication changes for each patient after first visit in Neurology clinic. Column indicates the addition or elimination of a medication, with medication dosing reductions represented by a 0.5 reduction

After the first appointment with Neurology clinic, only 3 patients of 25 were started on an additional pain medication, abortive or preventative migraine medication. The patients who were started on new medications were all seen in Neurology clinic within 6 months of traumatic brain injury. However, they did not significantly differ from the population in regards to duty status, comorbid conditions, or outcome measures. Six patients (24%) were titrated down or discontinued from prior medications. The other patients (68%) were maintained on their current number of medications (Fig. 2).

**Fig. 3:**
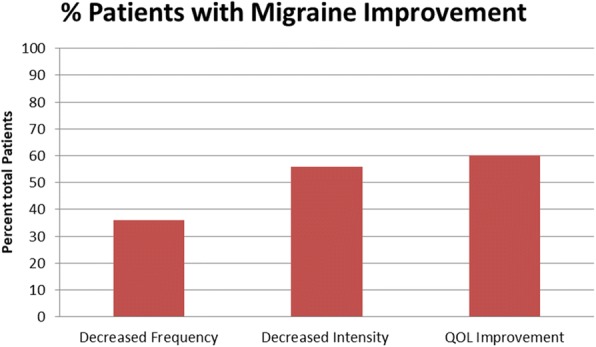
Percent of patients with migraine improvement

**Fig. 4:**
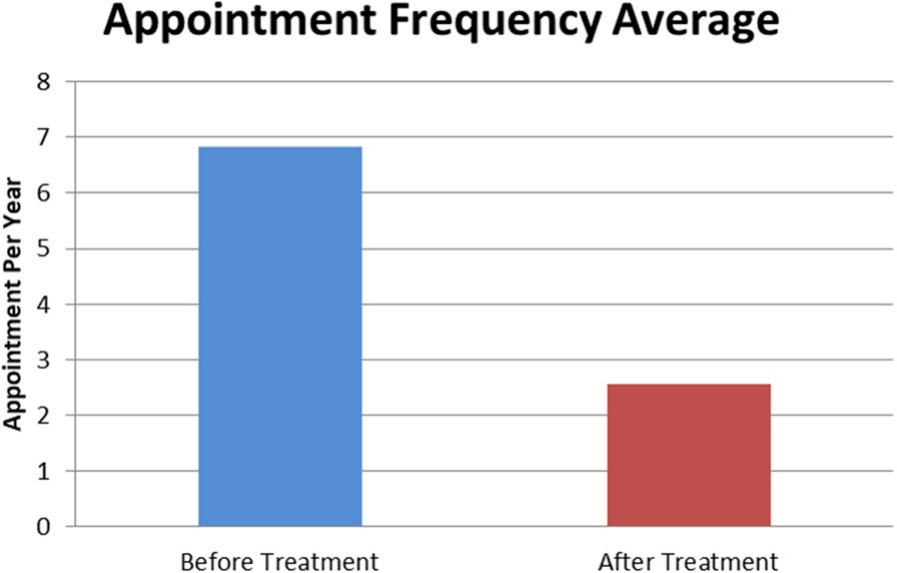
Average appointment frequency

**Fig. 5:**
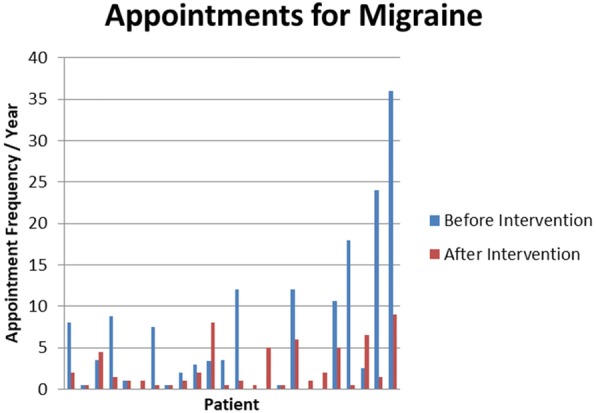
Individual appointment frequencies before and after intervention at Neurology clinic

After intervention, there was a 36% decrease in headache frequency and 56% decrease in headache severity within the PTHA / CPTHA cohort. Sixty percent of patients had reports of improved quality of life as compared to the 2 years prior to intervention (Fig. 3). Average appointments made per year for migraine or headache decreased from 6.8 to 2.6 after intervention, representing more than a 250% decrease in appointment frequency (Fig. 4). Of the cohort, 79% of patients had a decrease in appointment frequency (Fig. 5). The majority of patients (84%) were able to remain active duty and working in full capacity in Okinawa, Japan. Of the 25 patients treated for PTHA or CPTHA, four had a medical board initiated over the 2 years of evaluation and treatment, indicating they were no longer able to function in a military operational capacity. They were returned to the United States from Okinawa due to restrictions of job performance and to pursue further treatment. Two of the four patients were medically boarded for migraines, one for dysautonomia and one for a cardiac condition.

## Discussion

This data represented the first cohort published with benefit from a systematic approach to the treatment of PTHA and CTPHA. Despite the small sample size, the pilot study demonstrated reduction in migraine frequency, intensity, and duration (Fig. 3). Encounters, including outpatient appointments and emergency room visits, were also reduced, minimizing the resource utilization from PTHA/CPTHA treatment. Although it is suspected that a reduction in encounter visits demonstrated improvement in headache burden, this observation may have had other causes, including perception of limited utility or limited appointment availability. In future studies, scheduled phone calls may be useful to further investigate this cause.

In our cohort, nearly 90% of patients were prevented from increasing polypharmacy and nearly one quarter of patients were weaned or discontinued from medications (Fig. 2). However, since most patients were still taking medications and we did not compare to the natural history of PTHA/ CPTHA, this intervention of interest is less clear. Patients who sustained a mild traumatic brain injury with loss of consciousness were significantly more likely to have been started on 2 or more medications for headache prior to presentation to Neurology. They were also significantly more likely to have coinciding psychiatric comorbidities. The causal relationship is not known, and it is possible that the psychiatric comorbidities are what led to increased polypharmacy. However, it is also possible that providers are more willing to prescribe pain and headache medications in the setting mTBI with of loss of consciousness. Given unique efforts from our single Neurologist, medication reduction was believed to be more prominent in our cohort than in other CTPHA and PTHA populations. It was therefore considered an intervention that contributed to outcome measures. In future research, it would be useful to compare this treatment method to patients treated by other providers who did not emphasize polypharmacy reduction.

Outcome measures included migraine frequency, severity and duration. Data was obtained from the electronic medical record and assumes accurate and detailed documentation. Pain scales were based on pain at time of clinic visit or included in reference to pain at times of migraine. This may be altered due to the tendency to present to clinic during times of headache or increased pain. Quality of life was also assessed, which was self-reported and documented in medical records. In future research, we would standardize the form of data collection and utilize well-accepted outcome measures for migraines, including the headache-impact scale. Additionally, our data set assumes improved outcomes are due to intervention performed within the Neurology clinic. However, many patients were undergoing coinciding psychiatric treatment which may have introduced uncontrolled confounders. Psychiatric co-morbidities had a high prevalence among our cohort (64%) and the interplay between mental health and PTHA / CPTHA is widely recognized [33, 34]. Coinciding psychiatric treatments were encouraged, and could have altered our outcomes. Future studies would benefit from inclusion of psychiatry records and depression scales to better assess correlation between migraine improvement and improvement of psychiatric comorbidities.

In our cohort, 84 % of patients were able to remain active duty and deployable. Although there is no published comparison for the number of patients with PTHA/CTPHA who are medically separated from the military each year, this is an accepted success within the given population. These results are ultimately a marker of reduced separation from the military, limitations of duty, or return to the United States from a deployed, overseas setting.

Despite recognized benefits of this systematic approach, there were evident flaws in data collection and outcome measures. First, we recognize that this is a small sample size which increases the possibility for a skewed dataset. It favored a younger patient population and was primarily active duty, deployed service members. However, we hope this study can serve as a framework for future research with a larger population. In future research, we would further subdivide patients based on PTHA and CPTHA subtype and we would stratify by time from injury. We would recommend use of headache diaries in order to better assess headache subtype and possible triggers. Secondly, this was an observational study, with comparisons made among the same group before and after intervention. Without a control group, it is hard to assess the natural progression of headache after TBI and our intervention was not independently assessed. Even without intervention, we may expect an improvement in headache burden as time increases from traumatic brain injury [35–37]. In order to minimize the more significant early improvements, we started data collection greater than 1 month from inciting trauma. Future research may benefit from stratifying patient population based on time from traumatic brain injury.

There are no consensus guidelines or FDA-approved medications for management of PTHA or CPTHA. The lack of a systematic approach ultimately promotes polypharmacy, leading to increased disability and mortality in this population [6, 16, 28, 38–48]. Our study focused on hydration, lifestyle changes, and biofeedback in lieu of pharmaceuticals, which seemed to have an impact on reducing clinic visits and improving quality of life. Hydration has a well-known benefit for sufferers, but is a relatively new treatment strategy for PTHA and CPTHA [6, 22, 49, 50]. This study is the first to formally implement a hydration and lifestyle approach, and initial results in CPTHA and PTHA are promising.

## Conclusions

A combined multidisciplinary approach to treatment is likely to be most efficacious in the treatment of chronic post-traumatic headache and post-traumatic headache, especially in the military population given known lifestyle stressors and risk of TBI. Our goal was to improve quality of life and reduce disability, time away from work, hospitalizations, and polypharmacy in this population. This was made possible by having a central source of care, a single neurologist, and a collaborative effort with all providers through telemedicine in order to provide follow-up and continuity of care in a deployed setting in Okinawa, Japan.

This pilot study and its limitations will serve as a foundation for design of future research. The design and systematic approach will be used in a larger cohort, when we are able to stratify patients based on psychiatric comorbidities, more deliberate medication reduction, headache subtype and by time from initial traumatic brain injury. Our results can serve as a basis for instituting a systematic approach focused on lifestyle intervention, stress management and polypharmacy reduction in patients with post-traumatic headache and chronic post-traumatic headache. Flaws and shortcomings as documented above will be taken into account in future research, in order to enhance the credibility of the study and further illuminate the true effects of intervention. We believe that instituting such an approach on a larger scale would provide more definitive data on this approach for PTHA and CPTHA.

## Abbreviations

AD: Active duty
CBT: Cognitive behavioral therapy
CPTHA: Chronic post-traumatic headache
EMR: Electronic medical record
FDA: Federal Drug Association
HIPPA: Health Insurance Portability and Accountability Act of 1996
NSAIDS: Non-steroidal anti-inflammatories
PTHA: Post-traumatic headache
PTSD: post-traumatic stress disorder
QOL: Quality of life
TBI: Traumatic brain injury
